# Analysis of Sensitivity, Linearity, Hysteresis, Responsiveness, and Fatigue of Textile Knit Stretch Sensors

**DOI:** 10.3390/s19163618

**Published:** 2019-08-20

**Authors:** An Liang, Rebecca Stewart, Nick Bryan-Kinns

**Affiliations:** 1 School of Electronic Engineering and Computer Science, Queen Mary University of London, London E1 4NS, UK; 2 Dyson School of Design Engineering, Imperial College London, London SW7 2DB, UK

**Keywords:** wearable, e-textiles, smart textiles, strain sensor, stretch sensor

## Abstract

Wearable technology is widely used for collecting information about the human body and its movement by placing sensors on the body. This paper presents research into electronic textile strain sensors designed specifically for wearable applications which need to be lightweight, robust, and comfortable. In this paper, sixteen stretch sensors, each with different conductive stretch fabrics, are evaluated: EeonTex (Eeonyx Corporation), knitted silver-plated yarn, and knitted spun stainless steel yarn. The sensors’ performance is tested using a tensile tester while monitoring their resistance with a microcontroller. Each sensor was analyzed for its sensitivity, linearity, hysteresis, responsiveness, and fatigue through a series of dynamic and static tests. The findings show that for wearable applications a subset of the silver-plated yarn sensors had better ranked performance in terms of sensitivity, linearity, and steady state. EeonTex was found to be the most responsive, and the stainless steel yarn performed the worst, which may be due to the characteristics of the knit samples under test.

## 1. Introduction

Wearable devices have been developed to track the human body in a number of different areas, such as fitness [1], healthcare [2], entertainment [3,4], and fashion [5]. However, in early wearable computer research, the sensors and computing system were put into a pocket to be carried on the body [6]. Since the 1970s and 1980s, clothing, accessories, and even the body itself were developed as an interface for various analog and digital functions by researchers [7]. The exploration of digital textile interfaces provides the opportunity for wearable technology to be integrated into our daily lives and could provide access to data generated by the body.

More recently, with an increasing range of applications for wearable devices research into intelligent textiles has attracted considerable attention [8,9,10]. Clothing is regarded as a second layer of skin for the human body—it keeps our body warm and protects us from the environment. On the basis of enhancing the basic functionality of clothing, researchers have started to explore its connectivity and interaction with surrounding objects and environment. “Smart clothing” [11], “wearables” [12], and “wearable computing” [6] are innovating what otherwise would be generic clothing [13]. Electronic textiles (e-textiles) combine textile technology and wearable computing to give fabric new functionality [14]. Combined with wearable computing, it can be an information processing system that has the ability to sense, feel, react, and act through wearer’s subconscious or conscious actions and the surrounding environment [15]. Moreover, textiles provide enormous flexibility for system design with a wide range of fibers, yarn, fabrics, and manufacturing techniques can be developed to create products [15]. The development of textile sensors means wearable technology can move beyond handheld or pocket devices and be truly integrated into clothing [16,17,18].

One particular e-textile sensor that has attracted attention is the stretch or strain sensor. It has been used in an electronic sleeve for healthcare [19], in breathing monitoring applications [20], and to recognize upper body postures [21]. Stretch sensor properties are closely related to their fabrication and structure [22], and we are primarily interested in knit sensors that change their electrical resistance when under strain. For example, the number of wales (vertical knitted rows) and courses (horizontal knitted rows) of the conductive area, difference in structures like interlock or tubular knit, and also different wool blends and levels of stretch allowed by the fabric all feed into the sensor properties [23]. This follows on from earlier work by Metcalf et al. [24] who compared conductive yarn, knitting structures, and yarn compositions. They found that a single jersey knit structure with a silver-coated nylon yarn performed best for their sensor tracking knee movement. Later comparisons of knit sensors [25] found that the commercially produced Eeonyx fabric could be used to create more stable and less noisy sensors, but that other knitted fabrics may be preferable for applications of sensors that require a greater amount of stretch.

Whilst there are a wide range of applications for stretch sensors, our research is interested in using textile stretch sensors for dance applications, where the aesthetics and comfort of the sensors are highly valued alongside their sensing properties. There are several research areas where e-textiles may be used for dance applications, such as education [26] and new artistic expression [27]. Here, we examine how well the textile stretch sensors can detect movement with parameters similar to dance, focusing on materials that could be integrated at a later stage into a garment. Even though smart textiles sensors have been a subject of research for decades as outlined above, the literature has largely looked towards sports and medical applications. Textile sensors for dance movement and interactive art have not been examined extensively in a rigorous manner, though e-textiles have frequently been used in artistic works [28]. In this study, we evaluate the sensor properties of different conductive stretch fabrics in order to determine the most reliable sensor for dancer and computer interface design.

## 2. Materials

**Figure 1:**
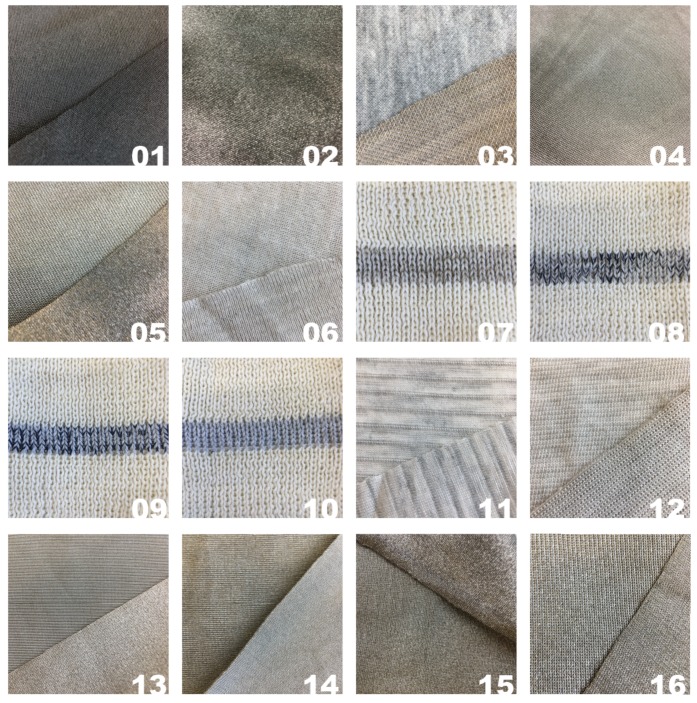
Photographs of the sixteen sensors along with their sensor number.

In this study, we compare twelve commercially produced conductive fabrics and four sensors knit in our research lab using two commercially available conductive yarns. These materials were chosen because they are commonly used by e-textile designers. The commercially produced knitted conductive fabrics have varying compositions, knit structures, and production methods which give the fabrics diverse properties. A full list all the sensors can be found in Table 1 with photos of each sensor in Figure 1.

### 2.1. Sensor Design

**Figure 2:**
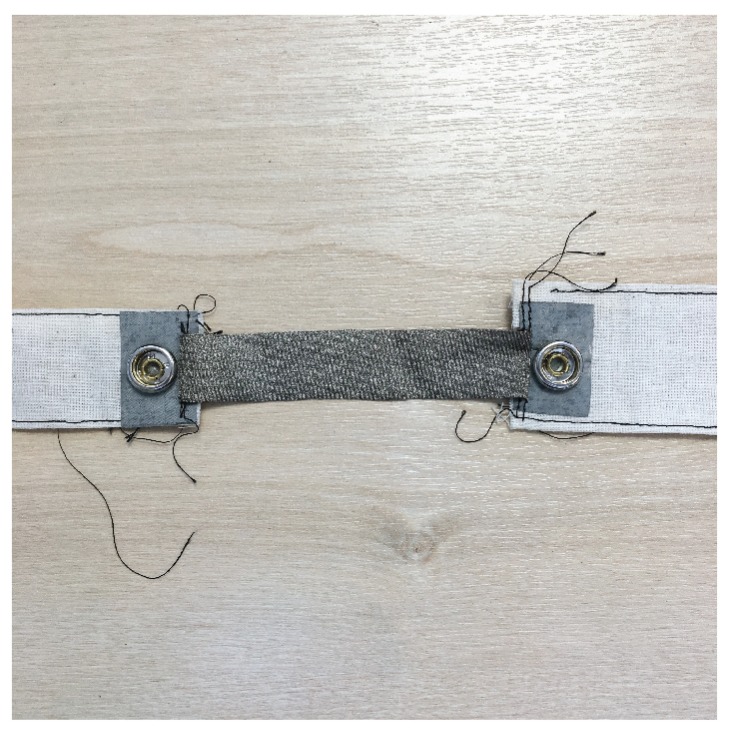
Sensor made with commercially produced conductive fabric.

Sixteen stretch sensors were constructed using the above materials. All the conductive fabrics were cut and the conductive yarns knit to the same dimensions of 20 mm × 100 mm when under no strain. As Figure 2 shows, each piece of conductive fabric is stitched to a piece of woven fabric. The woven fabric is attached to the tensile testing machinery during the stretching tests. Each end of the conductive sensor fabric is electrically connected to a press-fit snap via conductive ripstop fabric. The snaps provide a wired connection to a breadboard containing a microcontroller resistance measuring circuit.

The four sensors using commercially available conductive yarn (Samples 07 to 10) were knit in our research lab using a gauge 7 Dubied knitting machine (7 needles per inch) with a stitch tension of 9.5. The sensor is 6 rows in height, with a width of 30 needles on a double bed. All of the sensors start with 20 rows of white nonconductive wool (30/2 Nm Merino wool), then 6 rows of conductive yarn, then 20 rows of the white nonconductive wool again. Samples 08 and 09 are knit with blue elastane (Monofilament fiber: 100% Nylon, 70/23 × 2) throughout to enhance the elasticity and recovery. The same arrangement of conductive ripstop and snaps is used to connect the sensors to the microcontroller.

### 2.2. Conductive Materials

The sixteen stretch sensors each use one of three types of conductive materials: EeonTex; silver-plated yarn; and spun stainless steel yarn. Sample 01 is constructed from EeonTex, Samples 09 and 10 use spun stainless steel yarn, and all other samples are silver-plated yarn knitted sensors from commercially available fabrics or threads. Samples 07 to 10 are knit with a 1×1 rib knitting structure, Samples 03 and 06 have a double bed interlock knitting structure, all other sensors are knit with single bed jersey knit. Samples 07 to 10 were knit in our research lab using an industrial Dubied knitting machine to compare silver-plated and spun stainless steel yarns.

#### 2.2.1. EeonTex

Sample 01 is made from EeonTex LTT-SLPA, a conductive stretchable fabric produced by Eeonyx Corp. This material consists of 72% nylon and 28% spandex elastic fabric coated with a proprietary conductive polymer. It has a two-way stretch (warp and weft) and can be used for pressure and stretch sensor applications. This material is washable, but after 30 cycles of washing, there is no longer a significant change in resistance when the fabric is stretched or pressed [29].

#### 2.2.2. Spun Stainless Steel Yarn

The stainless steel sensors, Sample 09 and 10, were knit in our research lab using a spun stainless steel yarn (10/3 Nm) from Plug & Wear Srl. The spun yarn is 20% stainless steel and 80% polyester and consists of three threads plied together. The weight of the yarn is 10 meters per gram, resulting in a yarn count of 10/3 Nm.

#### 2.2.3. Silver-Plated Yarn

Silver is a popular material for conductive textiles as it has a low electrical resistance and is safe to be worn against the skin. It is most commonly plated around a nonconductive fiber which is then plied and woven or knit into a textile. There are many kinds of silver-plated materials in the market and Samples 02 to 08 and Samples 11 to 16 are silver-plated sensors with different material compositions and knit structures. Sample 02 uses MedTex 180, Sample 05 uses MedTex P-180, and Sample 15 uses Technik-tex P130B, all from Statex Produktions. Samples 11 to 14 and Sample 16 are from Chinese shielding fabrics manufacturer (DaZhi, QingDao, China). Sample 11 uses silver fiber antibacterial conductive cloth (DaZhi #3-17), Sample 12 uses metal fiber radiation protection fabric (DaZhi #3-2), Sample 13 uses 135 g 100% silver fiber four-way stretch fabric (DaZhi #3-18), Sample 14 uses 130 g 100% silver fiber four-ways stretch fabric (DaZhi #3-15), Sample 16 uses 100% silver fiber ion cloth (DaZhi #3-8-1). Samples 03, 04, and 06 are from Less EMF Inc. (Ithaca, NY, USA), with corresponding product numbers Cat.#1281, #A321, and #1224. In addition, Shieldex 110/34 dtex, 2-ply yarn from Statex Produktions is knit in Samples 07 and 08.

## 3. Methods

To evaluate the performance of the sensors for use in dance applications, we use two types of evaluation approaches to cover a range of movements that may occur in dance performance: (i) Evaluation of the sensors held in a static steady-state; and (ii) dynamic repeated movements of stretching and relaxing the sensors. An Instron 5900 Series Universal Testing Instrument (Instron, Wycombe, UK) was used to stretch the sensors.

To determine the maximum extension being measured, we recorded the maximum stretched length of the elbow joint bending on three people with different heights and genders (2 females, 1.60 m; and 1 male, 1.80 m). We found that the stretching range of the elbow joint was similar across our small sample—all around 60 mm. Accordingly, the extension value of tensile testing has been set as 60 mm.

### 3.1. Resistance Calculation

As the sensors under test all exhibit a piezoresistive effect, the resistance is the key parameter to observe. Therefore, we use a highly accurate measurement method to calculate the resistance changes of these sensors—the Wheatstone bridge circuit.

The Wheatstone bridge [30] is an electrical circuit that can accurately measure resistance. There are four resistors connected to the two legs of the bridge. Given the three known resistor values, we can calculate the unknown resistor’s value by balancing two legs of the bridge.

(1) $$R_{X}=\frac{\frac{R_{3}}{R_{1}+R_{3}}+\frac{V_{m}}{V_{b}}}{1-\frac{R_{3}}{R_{1}+R_{3}}+\frac{V_{m}}{V_{b}}}R_{2}.$$

In this study, we set up a Wheatstone bridge circuit for every sample. There are three known resistances, $R_{1}$, $R_{2}$, and $R_{3}$, with *Rx* being the stretch sensor. The value of $R_{x}$ is calculated by Equation (1), using an Arduino Uno microcontroller to read the voltage from the two sides of the bridge ($V_{1}$ and $V_{2}$), then by calculating $V_{m}$. The 5 volt supply voltage from the Arduino board is denoted at $V_{b}$. The values of $R_{1}$, $R_{2}$, and $R_{3}$ are dependent on the resistance of the sensor ($R_{x}$), so we measured the resistance of each sensor when stretched and released using a multimeter. The values for $R_{1}$, $R_{2}$, and $R_{3}$ are the mean of these two resistance values, rounded to the closest value of a commercially available resistor.

### 3.2. Dynamic Test

The samples are tested starting at 10% strain to ensure they are always under some tension. In the dynamic test, samples are stretched and released 100 times, from 110 mm to 170 mm, at a speed of 6 mm/s. The aim of this test is to evaluate the reliability of the sensors by analyzing the relationship between strain and the electrical resistance of the sensor, and whether that relationship remains consistent over multiple cycles. When looking towards dance applications, this will determine the extent to which the sensor could reliably detect body movement, such as the angular change of a joint like an elbow.

The tensile tester stretched the sensor and recorded the strain and extension exerted, also, in parallel, a separate microcontroller measured the electrical resistance. A sampling rate of 128 Hz was chosen as a sufficiently high rate as 120 Hz is the upper limit of motion capture cameras.

### 3.3. Static Test

The samples are initially stretched at a speed of 6 mm/s, then stopped and held at 70% strain for three minutes. Then, the samples are released at 6 mm/s and held for three minutes at 10% strain. This test examines how stable the sensor is over time, especially as the textile settles.

### 3.4. Data Analysis

The following metrics are extracted using the above data capture methods.

#### 3.4.1. Working Range and Gauge Factor

The working range of the sensors is the maximum and minimum electrical resistance values at the minimum and maximum stretch. Here, we measure each sensors working range as the maximum and minimum electrical resistance values of the sensor stretched from 10 mm to 70 mm.

Gauge factor (GF) is defined as the ratio of relative changes in electrical resistance (ΔR) to the mechanical strain (ε), described in the following equation:

(2) $$\mathrm{GF}=\frac{\Delta R/R}{\Delta L/L}=\frac{\Delta R/R}{\varepsilon },$$

where *R* is the initial resistance, ΔR is the change in resistance, *L* is the initial length, and ΔL is the change in length.

#### 3.4.2. Linearity

In this study, we look at the linear relationship between resistance and strain during stretching and relaxation in the dynamic test, where one hundred cycles of stretching and relaxation data are aggregated and the start and end of linear regions identified. In an ideal sensor, the resistance change should have a linear relationship to the extension so that the extension can be inferred from the resistance measurement.

**Figure 3:**
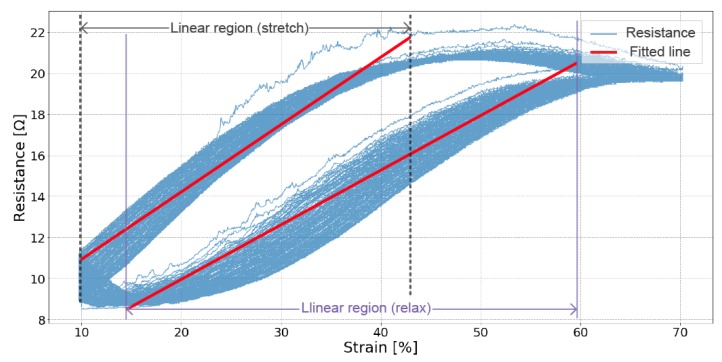
An example of linearity fitting of the dynamic test for Sample 04. The linear region of stretch and relaxation are identified and then fit with a line.

The best-fit line for the aggregated stretch and relaxation data is found and the root-mean-square error (RMSE) for that line is calculated. The sensor with the least error can be assumed to be the most accurate for measuring movement. Figure 3 illustrates the identification of the linear region and the fit line.

#### 3.4.3. Hysteresis

**Figure 4:**
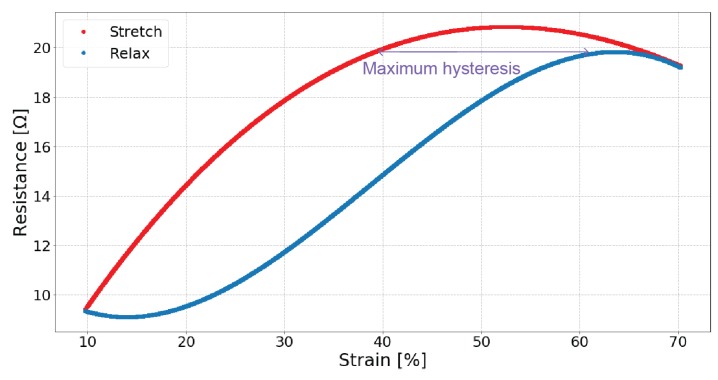
An example of the maximum hysteresis in the aggregated data from the dynamic test (Sample 04).

The dynamic test looks at the relationship between resistance and strain over repeated measures. The electrical resistance performs differently when the sensor is stretched compared to when it is relaxed, and the maximum difference between stretch and relax is the hysteresis. Figure 4 illustrates an example of the measured hysteresis. It is calculated by fitting a third order curve to the stretch and relaxation data, then calculating the maximum difference of the two curves.

#### 3.4.4. Fatigue

In the dynamic test, we examine the repeatability of the sensors over one hundred cycles, and fit the linear region of stretch and relaxation of the aggregate cycle data, then calculate the error of each stretch and relaxation cycle from that fit line. The difference of each cycle’s error compared with the sensor’s average error is examined to determine when fatigue first occurs.

#### 3.4.5. Responsiveness

**Figure 5:**
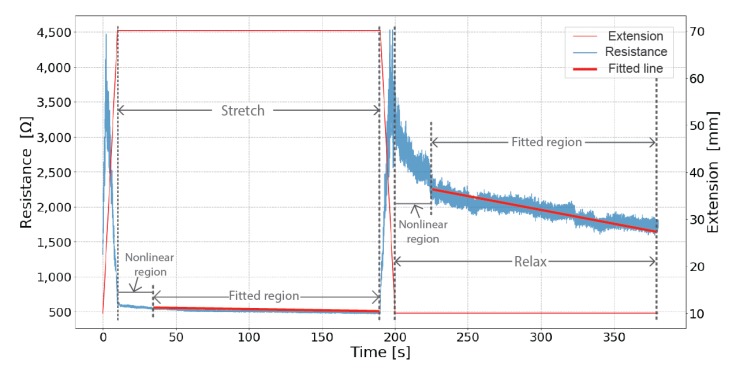
An example of linearity fitting a static test with Sample 9. The linear region is fit with a line when the sample is held in a stretched and relaxed position.

Responsiveness is defined as how fast the sensor responds electrically to a change in physical direction. We measure the response time when changing from stretching to relaxing and vice versa. We determine the nonlinear region by visual observation, then measure the length of time of the nonlinear region in dynamic and static tests. In the dynamic test, we measured the average nonlinear region of one hundred cycles in two directions, from relax to stretch and from stretch to relax. In the static test, we measure the nonlinear region when samples are initially held at 70% strain (stretched) and 10 strain% (relaxed), as shown in Figure 5.

#### 3.4.6. Steady State

The static test investigates each sensor’s performance when under constant strain. As Figure 5 shows, two parts of the static test data are examined: When the sensor is stretched to 70% strain and held for three minutes; and when relaxed to 10% strain and held for three minutes. For most of the sensors, there is a nonlinear region when the sensors’ state is first changed. The time until the linear region begins again is noted, then a line is fit to the subsequent region. The RMSE and the slope of the line are compared.

## 4. Results

### 4.1. Working Range and Gauge Factor

The working range of the sensors is the resistance range produced from under no strain, the unstretched length of 100 mm, to a stretched length of 170 mm. The initial electrical resistance of the sensors is measured using Rapid 318DMM Digital Multimeter before the subsequent tests to avoid fatigue influence the results. Table 2 shows the gauge factor as calculated from the sensor’s working range and resistance when under no strain.

Silver-plated sensors of Samples 05, 11, and 12 have relatively high initial electrical resistance values. Sample 05 had a large electrical resistance and produced such a noisy signal that we could not get valuable data from this sensor during the tests, therefore this sensor’s data is not analyzed in the following sections.

Spun stainless steel knitted sensors (Sample 09, 10) and one silver-plated sensor (Sample 14) have much larger gauge factors than the other sensors, almost over ten times higher than all the other sensors.

### 4.2. Linearity

We did not analyze the dynamic test data of the two sensors knit in our lab with conductive yarn, Samples 09 and 10. Their resistances became very large, passing almost no electrical current, after the completion of the dynamic test. This is possibly because they became deformed and lost their elasticity after being stretched one hundred times.

Table 3 summarizes the linearity performance of each sensor during stretching and Table 4 during relaxing. We identified each sensor’s linear region, the average resistance of the sensor while stretching, and the RMSE. Each sensor’s RMSE was divided by their average resistance ($\bar{R}$) to normalize the results.

**Figure 6:**
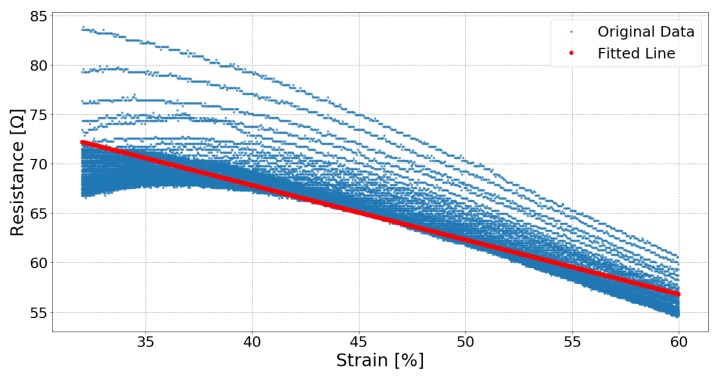
Sample 15, a silver-plated sensor (Technik-tex P130B), response and the fitted line from 32% strain to 60% strain while stretching.

**Figure 7:**
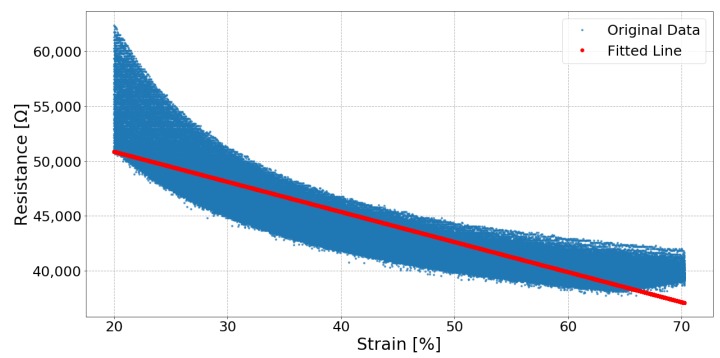
Sample 01 (EeonTex) sensor response and the fitted line while stretching between 20% strain and 70% strain.

**Figure 8:**
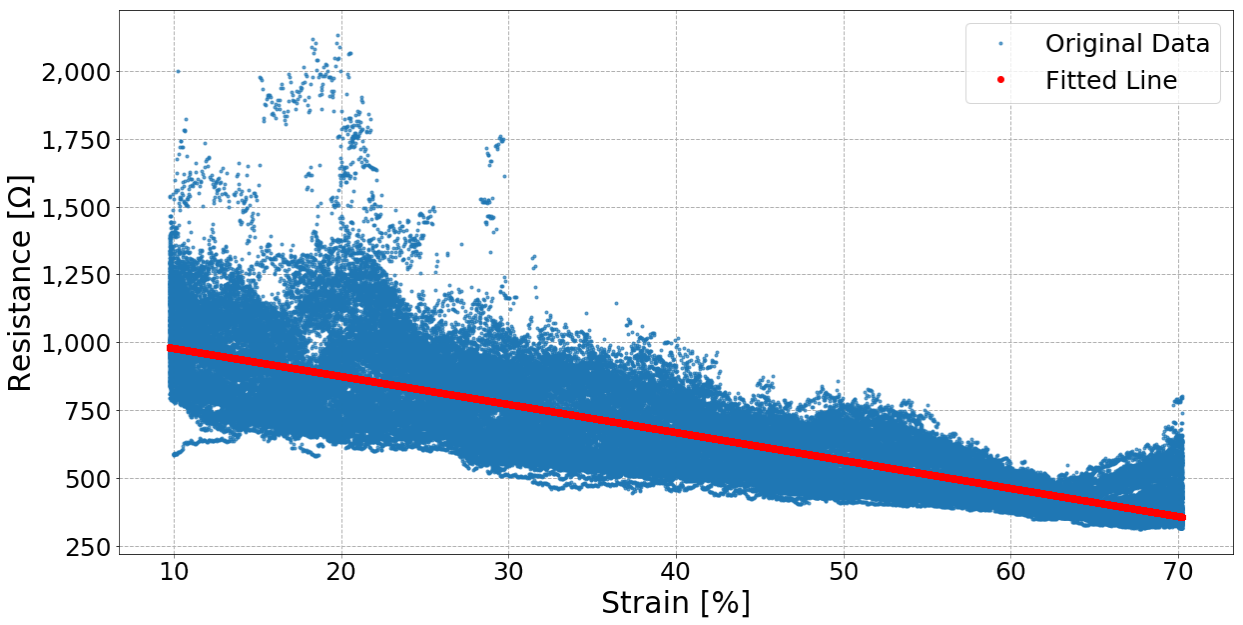
Sample 08, a lab-knitted sensor, response and fitted line of one hundred cycles of stretching between 10% strain and 70% strain.

Figure 6, Figure 7 and Figure 8 respectively show the result of Sample 15, Sample 1 and Sample 8. Each shows the output from one hundred stretches and identifies the linear region fit with a line. They show examples of a silver fabric, EeonTex, and a lab-knitted sensor and are also illustrative examples of the best performing (Figure 6), middle performing (Figure 7), and the worst performing (Figure 8) results.

### 4.3. Hysteresis

The maximum measured hysteresis for each sensor is shown in Table 5. Figure 4 can be referred to as an example plot of the hysteresis.

### 4.4. Fatigue

During one hundred cycles of stretching and relaxation, 9 of the sensors show resistance change and 10 of the sensors show signs of fatigue. With the increasing cycles, resistance of Sample 1 decreases and resistance of Samples 2, 3, 6, 7, 8, 11, 12, and 16 increases. Table 6 shows the number of cycles when sensors were fatigued as indicated by a change from decreasing to increasing error (RMSE).

**Figure 9:**
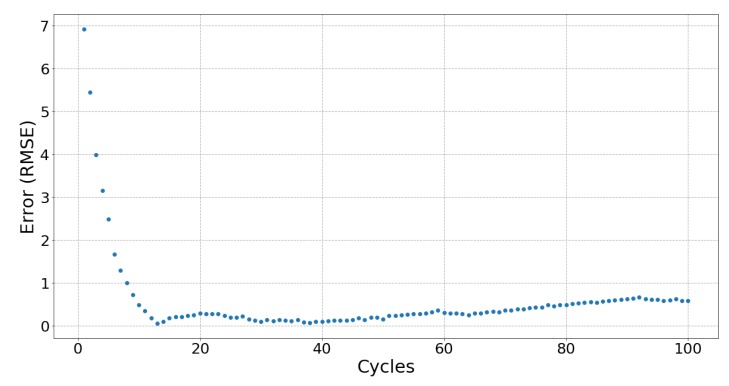
The error between each cycle of stretch and the fitted line of Sample 15. This shows the best repeatability result from all the samples‘ stretch and relax.

**Figure 10:**
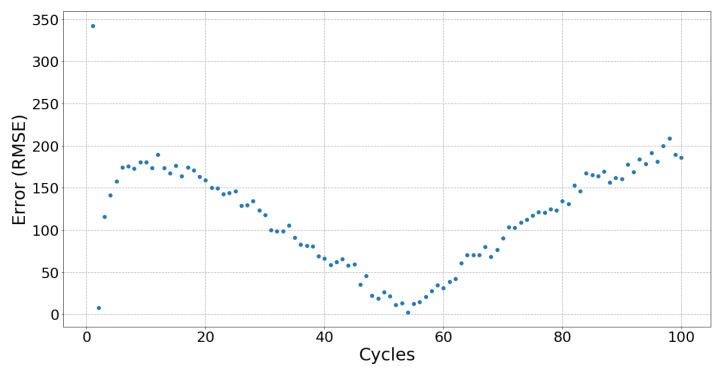
The error between each cycle of relaxation and the fitted line of Sample 01 (EeonTex). This plot shows the typical repeatability result from all the samples‘ stretch and relax. Most of the samples showed fatigue after 50 cycles of stretching.

**Figure 11:**
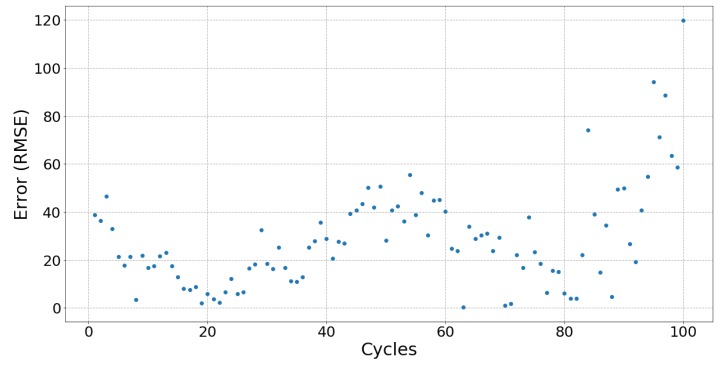
The error between each cycle of stretching and the fitted line of Sample 07. This plot shows a relatively worse repeatability result from all the samples‘ stretch and relax.

Figure 9, Figure 10 and Figure 11 respectively showed examples of each silver-plated fabric, EeonTex, and lab-knitted sensor’s response of one hundred stretch linear region and their fitted line, they are also the illustrative examples of the best (Figure 9), average (Figure 10), and worst (Figure 11) results.

### 4.5. Responsiveness

**Figure 12:**
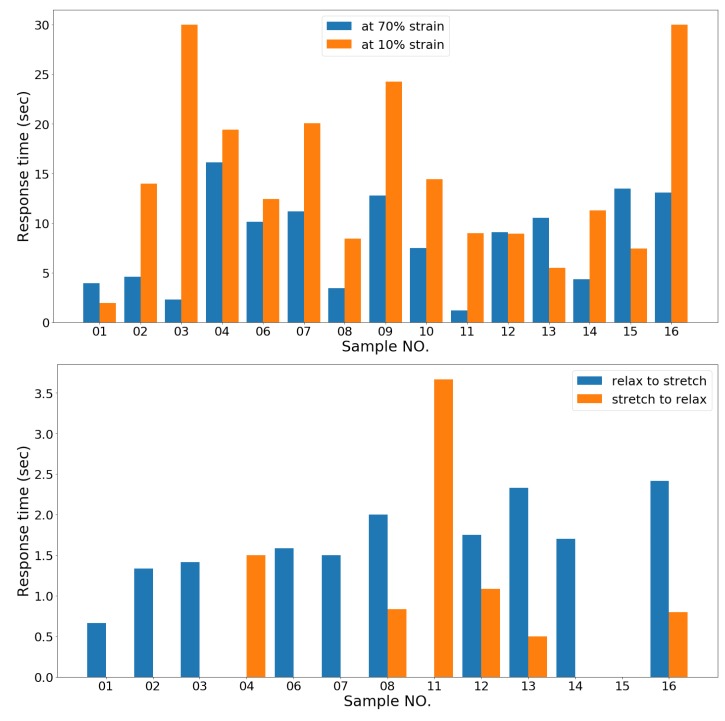
Comparison of each sensor‘s response time in static (**top**) and dynamic (**bottom**) tests.

In order to measure how quickly the sensors can respond to a change of movement direction, we calculate the time elapsed between a stopping or reversing of movement and when the sensor’s resistance returns to a linear output. Table 7 shows the response times when stopping at both 70% strain (sensors being stretched) and 10% strain (sensors being relaxed), and the sensor’s response time in dynamic tests when reversing from two direction—relaxing to stretching and stretching to relaxing. Figure 12 illustrates each of the response times.

### 4.6. Steady State

Table 8 shows each sensor’s information when they are held at a constant strain of 70%, the elapsed time of the nonlinear region, the average resistance value when the sensor is stretched, the RMSE of the fitted line, and the slope of the fitted line. The nonlinear time is how long the nonlinear region lasts at the beginning after the sensor is stretched and first held at 70% strain. As the sensors have different scales of resistance, their averaged resistance is calculated and used to normalize the RMSE for each sensor.

**Figure 13:**
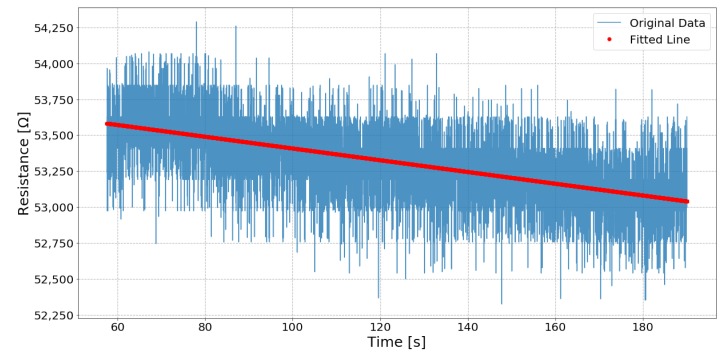
The fitting of Sample 1 while the sensor is at 70% strain. The EeonTex is a representational average result of all the samples.

Figure 13 shows an example of the line fitting, which is the average result of sensors held at a constant strain of 70%, and Table 9 shows each sensor’ analysis when held at a constant strain of 10%. Samples 16 and 03 are too noisy to fit a meaningful line.

## 5. Discussion

### 5.1. Working Range and Gauge Factor

The working range and gauge factors are important starting points when considering a sensor for a given application, such as capturing the movement of a dancer’s body. Sensors with larger gauge factors are more suitable for a variety of measurement locations on the body, especially along a joint where large angular movements need to be tracked. However, sensors with a small gauge factor may still be useful for movements where less fine-grained measurements are sufficient. When considering only the gauge factor, the two sensors with stainless steel yarn, Samples 09 and 10 appear to perform the best, but this is the only metric in which they perform well. They have a large working range, likely because of the structure of the spun conductive yarn which exhibits piezoresistivity even before being placed in the knit structure. With the exception of Sample 14, whose exact materials are unknown, all the silver-plated sensors have a similar gauge factor ranging from 0.87 to 2.97. The EeonTex, Sample 01, falls roughly in the middle of that range at 1.22. However, the gauge factor alone gives very limited insight into the performance of a sensor—further analysis is needed.

### 5.2. Linearity

An ideal sensor has a linear relationship between the input and output—in this case, the extension of the sensor ideally has a linear relationship with the electrical resistance of the sensor. This was evaluated by identifying what region of the sensor output could reasonably be considered linear, then fitting a line to that region and calculating the resulting error. The linear region was identified by manually inspecting the aggregated 100 cycles of stretching the sensor and 100 cycles of relaxing the sensor.

There is not a clearly identifiable sensor with the most ideal linearity performance as no single sensor had the largest linear region and also the least error when being stretched and relaxed. However, Samples 01, 03, and 15 all performed well in aggregate across these metrics. For example, Sample 15 was found to have the least error compared to its fit line when being stretched, but its linear region is only from 32% strain to 60% strain, which is a smaller range than most of the other sensors. Sample 03 has a slightly larger error than Sample 15 when stretched, but a much larger linear region from 18% to 70% strain. Therefore, when using the sensors to track movement, Sample 15 would predict the movement more precisely but for a limited range of motion. Sample 03 would track almost the entire range of a movement up to 70% strain, but with less precision.

Two of the lab-knitted sensors, Samples 09 and 10, were discarded during these tests as their resistances became so large as to approximate not passing any electrical signal. This is likely related to the sensors becoming significantly deformed and losing their elasticity after one hundred stretching cycles. The other pair of lab-knitted sensors with silver-plated yarn, Samples 07 and 08, also produced noisy signals probably due to their relatively loose knit structure.

Generally, the sensors‘ RMSE measurements are larger when relaxing than when stretching. We propose this is because, during stretching, the sensor’s resistance changes with an external force consistently being applied, so the sensor’s performance is relatively stable. However, when being relaxed, there is no longer an external force being applied, so the performance of the sensors is fully dependent on the material’s ability to rebound. Therefore, the relaxation also reflects the elasticity of the sensor‘s material and structure.

Measuring the hysteresis during stretching and relaxation quantifies the differences between the two movement directions, with an ideal sensor containing no hysteresis. Sample 01, EeonTex, has the smallest amount of hysteresis with the silver-plated sensors having diverse values ranging from 13% to 29%. The more-elastic sensors tend to have the lowest hysteresis. The lab-knitted sensors have the largest hysteresis of around 30%, indicating that the lab-knitted sensors take longer to recover. They are knit at a significantly larger gauge than the commercially manufactured fabrics, which may also play a role.

### 5.3. Fatigue

When used in applications such as tracking body movement, sensors are stretched more than once, meaning that fatigue may affect the performances of a sensor over time. Only two sensors, Samples 03 and 16, did not show signs of fatigue after 100 cycles of stretching and relaxing, with Sample 15 showing no signs of fatigue during stretching but did after 75 relaxation cycles. Figure 9 shows Sample 15 with no fatigue during stretching as the error is kept consistent, unlike Figure 10, which shows a trend typical for most of the sensors with errors increasing after 75 cycles. For most sensors, there appears to be a “breaking in” period (around 10 to 20 cycles) when there is greater error in the beginning of the cycles which then gradually stabilizes. The exceptions are the lab-knitted sensors, seen in Figure 11, which are less stable overall until about 80 cycles when fatigue sets in.

### 5.4. Responsiveness

The ideal performance of a stretch sensor has an identical linear performance during both stretching and relaxation and can instantaneously transition between these two states. This is particularly important in body movement when tracking, for example, a joint like an elbow opening and closing. In reality, this is not physically possible to do absolutely instantaneously due to the material constraints, but not all materials have the same responsiveness.

In the static test, no sensor has a linear performance instantly when the sensor stops moving at both 70% strain and 10% strain, but each sensor has a different response depending on whether the sensor stopped after being stretched or released. As Table 7 shows, in the static test, most of the sensors take less time return to linear performance when it has been stretched and held at 70% strain, likely because there is an external force stretching the sensors and keeping them stable. Among all the samples, Samples 01, 03, and 11 were ranked with the quickest response times when held at 70% strain. However, Samples 01, 13, and 15 have quicker responses when they are stopped after relaxation than after stretching, which indicates these three samples have good elasticity which rebound when the sensors have been released. As Figure 12 shows, Sample 01, EeonTex, is the most responsive in general in the static test.

In the dynamic test, the sensors have quicker responses than in the static test as the sensors are kept constantly moving, but some of the sensors still have nonlinear regions when their movement reverses. The results show that the sensors respond more quickly when transitioning from a stretching state to a relaxing state. We propose this is because after several cycles of stretching and relaxation, most of the sensors are deformed, causing an increase in the length of the sensors, so the sensors are not under tension at the beginning of the cycle. Figure 12 shows Sample 04, 11, and 15 have effectively instantaneous response times when the samples change direction from relaxing to stretching. This indicates that these sensors have good elasticity and are not deformed after several cycles of stretching. When examining the change from stretching to relaxing, most of the sensors have effectively instant response times. Sample 15, a silver-plated sensor, has 0 s response times in both directions of the dynamic test so it is the best sensor for dynamic motion tracking.

### 5.5. Steady State

When held under steady tension, an ideal sensor outputs a static value. However, a textile sensor changes and settles over time with several factors influencing this behavior.

A line fit to the linear region of a sensor under constant strain ideally has a slope of 0. When held at 70% strain, Sample 14 performed the best under that metric, but was slightly noisier than Sample 13 which had the third best slope. Sample 13 was also the least noisy under a constant 10% strain, though Sample 04 had the best slope under that condition.

We found the error and fitted line slope is larger when the sensors are relaxed, likely because the sensors have no external force being applied and instead rely entirely on their own elastic rebound during the relaxation. The more-elastic sensors have a more stable performance, such as Sample 15 which has the best result when relaxed. It has slightly larger resistance and RMSE than Sample 13 but shows better fitted line slope, which means Sample 15 has better elasticity and stability when relaxed.

Combining the results of sensors held at 70% and 10% strain, we found the sensors which have small average resistances are less noisy and also have better a slope. Samples 13 and 15 performed better in the static test in general as they are less noisy and have the smallest slope. Sample 01, the EeonTex, has a larger error and slope, especially when stretched. Surprisingly, it performed worse than all the lab-knitted sensors.

The steady state of the sensors would reflect how well the sensors will present the pause in the middle of dance movements. For example, when a dancer bends their elbow and keeps the position for a while, then changes to another position. The sensors which have small resistance and better elasticity will be able to sense these kinds of movements in real time.

## 6. Conclusions

This study compared sixteen stretch sensors constructed from commercially available materials, evaluating metrics that best align with the desired characteristics of wearable sensors for tracking body movement. We concentrated on different aspects of the linearity of the sensor responses and how they respond in dynamic motions, looking at sensitivity, hysteresis, responsiveness, and fatigue. We found the sensitivity or gauge factor of a given sensor was a poor predictor of how it would perform in subsequent tests. What is more important is the choice of materials and structure, with commercially knit silver-plated yarn at a tight gauge yielding the most consistent success.

Overall, we found Samples 13 and 15 to have the best general results, especially in respect to linearity. Both of the samples are silver-plated yarn, single bed jersey knits. They have relatively small working range, so they would be good to use for small-scale body movement tracking such as in the muscle, back, and spine. EeonTex has the least hysteresis and fastest responsiveness, so it is particularly suited to rapid, dynamic movement tracking. When compared to the commercially produced fabrics, the lab-knitted sensors did not work well, especially the stainless steel sensors. Among the four lab-knitted sensors, the samples knitted with silver yarn performed better than the stainless steel samples, though the addition of elasticated yarn had mixed results. The addition of elastic only improved the responsiveness and not the other metrics. However, all of the lab-knitted sensors had significantly looser gauges that likely contributed to their poor performance. With the identification of materials and structures that do perform better, future work can encompass recreating those sensor characteristics directly into knit garments.

This study only examined a small selection of characteristics under controlled settings. Before these sensors can be robustly used in real-world applications, future work around the effect of sweat and body heat along with considerations regarding washing, color, texture, and maintenance is needed.

## Figures and Tables

**Table 1:** Summary of samples.

Sample No.	Composition	Knit Structure	Description
01	Nylon 72%/Elastan 28%	single bed jersey knit	EeonTex
02	Nylon 94%/Elastomer 6%	single bed jersey knit	Silver-plated
03	Silver fiber 35%/Cotton 40%/Polyester 25%	double bed interlock	Silver-plated
04	Nylon 76%/Elastic fiber 24%	single bed jersey knit	Silver-plated
05	Nylon 94%/Elastomer 6%	single bed jersey knit	Silver-plated
06	Silver/Nylon 16%/Rayon 84%	double bed interlock	Silver-plated
07	silver-plated yarn	1×1 rib	Silver-plated
08	silver-plated yarn/Elastic yarn(100%nylon)	1×1 rib	Silver-plated
09	Spun stainless steel yarn/Elastic yarn(100%nylon)	1×1 rib	Spun stainless steel
10	Spun stainless steel yarn	1×1 rib	Spun stainless steel
11	Silver fiber/unknown	single bed jersey knit	Silver-plated
12	Silver fiber 24.6%/Cotton 36.8%/Polyester 38.6%	single bed jersey knit	Silver-plated
13	Nylon Spandex	single bed jersey knit	Silver-plated
14	100% Silver fiber/Nylon spandex	single bed jersey knit	Silver-plated
15	Polyamide 78%/Elastomer 22%	single bed jersey knit	Silver-plated
16	100% Silver fiber/unknown	single bed jersey knit	Silver-plated

**Table 2:** Working range and gauge factor of sensors.

Sample No.	Initial Resistance (Ω)	Working Range (Ω)	Gauge Factor
01	55,857	40,715	1.215
02	727.62	1297.6	2.972
03	8.36	4.34	0.866
04	12.92	20.22	2.608
05	13.87 M	12.29 M	1.477
06	20.86	16.43	1.313
07	667.48	902.92	2.255
08	565.11	1309.77	2.318
09	225.30	3706.36	27.418
10	771.43	6822.09	14.739
11	14.92 M	15.79 M	1.763
12	352.82 K	489.68 K	2.313
13	39.80	24.29	1.018
14	8.40	56.82	11.273
15	62.73	56.40	1.498
16	4.25	3.40	1.333

**Table 3:** Each sample’s linearity performance while stretching (ranked by root-mean-square error (RMSE)). Samples 05, 09, and 10 did not produce usable data, so were excluded from the analysis.

Sample No.	Linear Region (Strain/%)	Stretching $\bar{R}$ (Ω)	RMSE/$\bar{R}$
15	32–60	62.401	0.0280
03	18–70	7.399	0.0337
16	20–59	4.833	0.0382
13	23–70	38.777	0.0394
01	20–70	43,942.283	0.0479
04	10–42	16.377	0.0496
06	18–58	16.773	0.0626
02	30–70	284.615	0.1061
11	10–70	15,555,332.216	0.1084
14	28–70	9.827	0.1316
07	10–70	533.365	0.1933
08	10–70	669.435	0.1977
12	10–70	803,969.899	0.9825
05	–	–	–
09	–	–	–
10	–	–	–

**Table 4:** Each sample’s linearity performance while relaxing (ranked by RMSE). Samples 05, 09, and 10 did not produce usable data, so were excluded from the analysis.

Sample No.	Linear Region (Strain/%)	Relaxing $\bar{R}$ (Ω)	RMSE/$\bar{R}$
13	36–70	37.609	0.0353
01	20–70	45,676.049	0.0368
03	20–70	8.080	0.0437
16	10–60	5.396	0.0463
15	41–70	62.763	0.0479
14	28–70	9.048	0.0512
04	14.7–57	14.367	0.0544
06	14.6–70	18.517	0.0599
11	10–70	16,945,000.200	0.1239
02	30–70	419.864	0.1909
07	10–70	807.640	0.3041
08	10–70	995.857	0.3201
12	10–70	1,407,711.161	0.9507
05	–	–	–
09	–	–	–
10	–	–	–

**Table 5:** Maximum hysteresis error of the sensors.

Sample No.	Maximum Hysteresis (Approx.)
01	7%
03	13%
11	15%
12	15%
02	16%
15	17%
14	21%
04	24%
13	25%
16	27%
07	28%
06	29%
08	32%
05	–
09	–
10	–

**Table 6:** The cycle number at which sensors showed signs of fatigue.

Sample No.	Stretch (Cycles)	Relaxation (Cycles)
01	82	52
02	80	77
03	0	0
04	77	77
05	–	–
06	79	73
07	81	58
08	72	77
09	–	–
10	–	–
11	88	89
12	79	74
13	79	90
14	94	71
15	0	75
16	0	0

**Table 7:** Sensors’ response time.

Sample No.	At 70% Strain (s)	At 10% Strain (s)	Relax to Stretch (s)	Stretch to Relax (s)
01	3.936	1.919	0.667	0
02	4.568	13.989	1.333	0
03	2.304	180+	1.417	0
04	16.102	19.389	0	1.500
05	–	–	–	–
06	10.125	12.430	1.583	0
07	11.185	20.039	1.500	0
08	3.413	8.45	2.000	0.833
09	12.787	24.26	–	–
10	7.484	14.400	–	–
11	1.17	8.959	0	3.667
12	9.067	8.939	1.750	1.083
13	10.504	5.5	2.333	0.500
14	4.328	11.269	1.700	0
15	13.494	7.449	0	0
16	13.066	180+	2.417	0.800

**Table 8:** Summary of sensors held at 70% strain (ranked by slope).

Sample No.	Stretched $\bar{R}$	RMSE/$\bar{R}$	Fitted Line Slope
14	7.380	0.0024	0.00000178
16	3.404	0.0039	−0.00000354
13	26.437	0.0019	−0.00001151
04	19.539	0.0029	−0.00001853
15	48.376	0.0021	−0.00002187
07	264.348	0.0067	−0.00040201
12	239.792	0.0831	−0.00088288
03	6.169	0.0032	−0.00093813
09	500.327	0.0134	−0.00243984
10	749.237	0.0117	−0.00277595
06	13.715	0.0029	−0.00454466
02	108.481	0.0027	−0.00808992
08	307.380	0.0325	−0.03222228
01	53,311.627	0.0034	−0.03268672
11	6,851,052.653	0.0104	−15.50951161
05	–	–	–

**Table 9:** Results of sensors held at a constant strain of 10% (ranked by slope).

Sample No.	Relaxed $\bar{R}$	RMSE/$\bar{R}$	Fitted Line Slope
16	4.736	0.0375	−0.00000571
03	8.690	0.0162	0.00000706
04	9.307	0.0028	0.00001066
15	32.456	0.0022	−0.00001234
13	24.834	0.0021	−0.00003154
14	8.431	0.0069	−0.00003731
06	16.689	0.0037	−0.00004653
07	665.198	0.0142	−0.00019367
02	452.813	0.0040	−0.00144364
08	616.229	0.0111	−0.00178679
10	2239.344	0.0118	−0.01216064
09	1933.900	0.0274	−0.02570112
01	102,725.338	0.0080	−0.33534839
12	104,871.145	0.0139	−1.24070950
11	16,616,606.761	0.0143	−28.49458985
05	–	–	–
